# Effectiveness of low-dose radiation therapy in COVID-19 patients globally: A systematic review

**DOI:** 10.12688/f1000research.74558.1

**Published:** 2022-01-19

**Authors:** Sirish Raj Pandey, Saroj Adhikari Yadav, Swotantra Gautam, Kalpana Giri, Anirudra Devkota, Shipra Shrestha, Shreya Bhandari, Santosh Baniya, Bibhuti Adhikari, Bibek Adhikari, Shila Neupane, Jenish Bhandari

**Affiliations:** 1 Medical Oncology, Nepal Cancer Hospital and Research Center, Lalitpur, Province 3, 44700, Nepal; 2 Patan Academy of Health Sciences School of Medicine, Kathmandu, 44600, Nepal; 3 B.P. Koirala Institute of Health Sciences, Dharan, 44705, Nepal; 4 B.P. Koirala Cancer Hospital, Chitwan, 44204, Nepal; 5 Patan Academy of Health Sciences, Kathmandu, 44600, Nepal; 6 Shahid Gangalal National Heart Center, Kathmandu, 44600, Nepal; 7 Metro City Hospital PVT Limited, Pokhara, 33700, Nepal; 8 Aamda Hospital Damak, Damak, 56604, Nepal; 9 All Nepal College of Medical Education, Kathmandu, 44600, Nepal

**Keywords:** COVID-19 pneumonia, CT-scan, low-dose radiation therapy, LDRT, systematic review

## Abstract

**Background:** Novel Corona Virus Disease 2019 (COVID-19) can affect multiple organs, including the lungs, resulting in pneumonia. Apart from steroids, other anti-COVID drugs that have been studied appear to have little or no effect on COVID-19 pneumonia. There is a well-known history of inflammatory disease, including pneumonia, treated with low-dose radiation therapy (LDRT). It reduces the production of proinflammatory cytokines, Interleukin-1a (IL-1a), and leukocyte recruitment.

**Methods: **A comprehensive literature search was conducted using PubMed, Scopus, Embase, CINAHL, and Google Scholar, with keywords such as “radiotherapy,” “low-dose radiation therapy,” “low-dose irradiation,” “covid-19 pneumonia,” “SARS-CoV-2 pneumonia,” and “covid pneumonia.” with additional filters for human studies and customized articles in accordance with the Preferred Reporting Items for Systematic Reviews and Meta-Analyses. We reviewed randomized controlled trials, quasi-experimental studies, cohort, case-control, and cross-sectional studies with a clearly defined intervention, including low-dose radiotherapy alone or in combination with any therapy to treat COVID-19 pneumonia from December 2019 to May 2021. Patients receiving standard or high-dose radiotherapy, including for other diseases, were excluded. Zotero software was used to collect and organize research from various databases, remove duplicates, extract relevant data, and record decisions. Participants’ demographics and baseline status were obtained from the full-text articles along with the intervention’s outcome/effect on patient status.

**Results:** Four studies with 61 participants that met the inclusion criteria were included. One was a double-blind randomized controlled trial, one a non-randomized trial, while the other two were single-arm clinical trials. Low-dose radiation therapy did not show any significant improvement in COVID-19 patients.

**Conclusion: **Only two studies included in this review demonstrated an improvement in inflammatory markers; however, patients were also given steroids or other drugs. Therefore, the confounding effects must be considered before drawing conclusions. This systematic review does not support mortality benefit, clinical course improvement, or imaging changes with LDRT.

## Introduction

The novel coronavirus disease caused by SARS-CoV-2 (severe acute respiratory syndrome coronavirus 2) has led to global catastrophe since December 2019. ^1^ Mostly COVID-19 (coronavirus disease 2019) patients are asymptomatic or present with mild to moderate symptoms. Some patients, however, may present with severe symptoms and quickly deteriorate to end-organ failure or acute respiratory distress syndrome (ARDS). ^2^^,^^3^ The ICU (Intensive Care Unit) settings management has shown improvements in the survival of patients. ^4^ Still, the management has remained primarily supportive, and more intervention and treatment options are required for severe and critically ill patients in ICU. ^5^ Studies have shown diffuse alveolar damage with inflammatory infiltrates in postmortem analysis of COVID-19 patients, which compromises the gas exchange. ^6^ The mortality rate among critically ill patients in ICU still remains as high as 30–40% globally. ^7^ Mortality up to 80% is seen once a COVID-19 patient is dependent on mechanical ventilation. ^8^^–^^10^ ARDS leading to respiratory failure is the most common cause of mortality among COVID-19 patients. ^11^

Viral pneumonia can result in systemic inflammation and multiorgan failure due to cytokine storms caused by a severe inflammatory response in the body. ^12^ Such excessive host immune response and direct viral damage can not only cause significant lung injury and diffuse alveolar damage but also have a reaction like local microvascular thrombosis and raised inflammatory markers. ^6^^,^^13^ Remdesivir and other studied antiviral drugs did not significantly affect overall mortality, ^5^ the World Health Organization has recommended not using these drugs based on the latest studies. ^14^

One novel approach suggested for COVID-19 patients is the whole-lung LDRT (low-dose radiation therapy). ^15^ LDRT has anti-inflammatory properties like lowering proinflammatory cytokine levels (e.g., Interleukin 1a (IL-1a)) and inhibiting the recruitment of leukocytes. ^16^^–^^19^ Hence, since the 20th century, LDRT has been used to manage inflammatory disorders, including pneumonia, with several studies showing potential benefits. ^15^^,^^20^ The evidence of management of viral pneumonia in the past has led to the proposal of LDRT as a possible intervention to manage COVID-19 pneumonia. ^21^^,^^22^ Numerous prior studies have described the mechanism of how LDRT can provide a therapeutic advantage. ^14^^,^^23^^–^^29^ However, the efficacy of LDRT is not well studied for the treatment of COVID-19 pneumonia. ^5^ Because of the limited treatment options for COVID-19 and the minimal risk of toxicity, several clinical trials of LDRT for COVID-19 management are being carried out with 0.3 to 1.5 Gy (Gray) radiation doses. ^30^^,^^31^

We conducted a systematic review to evaluate the clinical and radiological effects of LDRT in patients with severe acute respiratory syndrome (SARS) due to COVID-19.

## Methods

### Protocol registration

This systematic review was performed to analyze the effectiveness of LDRT for COVID-19 pneumonia patients globally. This review was registered on PROSPERO on 6 ^th^ March 2021 (CRD42021258776).

### Eligibility criteria

These criteria were sought for inclusion in the study:

A) Population: COVID-19 patients globally.
B) Intervention: Low dose radiotherapy/low dose radiation therapy with any combination of treatment for COVID-19 pneumonia.
C) Comparison with a control group in the study.
D) Outcomes: Improvement in lung consolidation (chest X-ray and CT scans) and inflammatory markers (level of cytokines, I.L./Interleukins, e.g., IL6), O _2_ saturation levels, C-reactive protein (CRP))
E) Study Design: Randomised controlled trial (RCT), cohort, cross-sectional, case-control, quasi-experimental, case studies.

We included randomized controlled trials (RCTs), quasi-experimental studies and cohort, case-control, cross-sectional studies with a clearly defined intervention published between December 2019 and May 2021. We excluded case reports, reviews, perspective/opinion articles, newspaper articles, book chapters/medical books.

### Data extraction

Twelve reviewers conducted study selection (SRP, SB ^1^, AD, JB, SB ^2^, SS, Bibh. A, KG, SG, SAY, Bibe. A, SN). Zotero software (version 5.0.96.2) was used to assemble and organize the studies obtained through the various databases. All ten reviewers then screened the titles and abstracts of the studies in four groups, with two members in each group. Duplicate studies were removed. Five reviewers, SRP, SB ^1^, AD, JB, and SB ^2^, independently screened records for inclusion. Four reviewers, SRP, SS, Bibh. A, and KG, checked the decisions. We included human studies involving both sexes in which patients received low-dose radiation therapy to treat COVID-19 pneumonia. The risk of bias and quality of studies was assessed by the Cochrane risk-of-bias tool for randomized trials version 2 (RoB 2), Risk of Bias in Non-Randomized Studies - of Interventions (ROBINS-I), and the National Institute of Health (NIH) quality assessment tools for before-after studies with no control group. Any confusion or disagreements were resolved among all the members. Four reviewers, SRP, SG, SAY, and KG discussed the results and prepared the first draft of the manuscript, and five reviewers, SRP, SAY, SG, Bibe. A and SN reviewed subsequent edits. The full-text articles were screened to obtain the following data: author, year of publication, study design, study setting, participant demographics and baseline status, type, duration and times of intervention, and outcome/effect on the patient status following the intervention. We also extracted the primary outcomes, which were the number of deaths or discharges following the intervention, and the secondary outcomes, which were the oxygen status of the patients, CT-scan changes, and requirement for mechanical ventilation following the intervention. We also determined any toxicity of the intervention and changes in inflammatory markers.

### Search strategy

A comprehensive literature search was performed on the following databases: PubMed, Scopus, Embase, CINAHL (Cumulative Index of Nursing and Allied Health Literature), and Google Scholar using relevant Medical Subject Headings (MeSH) and keywords termed “radiotherapy,” “low-dose radiation therapy,” “low-dose irradiation,” “covid-19 pneumonia”, “SARS-CoV-2 pneumonia”, “covid pneumonia”, and “coronavirus pneumonia” with additional filters of human studies and English language (Please see underlying data ^32^). Preferred Reporting Items for Systematic Reviews and Meta-Analyses (PRISMA) guidelines were used to search for the studies which evaluated the role of the effectiveness of low-dose radiation therapy for COVID-19 pneumonia patients globally. Five reviewers, SRP, SB ^1^, AD, JB, and SB ^2^, independently screened records for inclusion. Four reviewers, SRP, SS, BA, and KG checked decisions to include articles. In case of dissent, all the reviewers re-evaluated the inclusion and exclusion criteria, and the final decision was made based on the majority’s judgment. The PRISMA checklist 2020 was followed throughout the process. ^32^

### Risk of bias assessment

For the RCTs, we used the Cochrane risk-of-bias tool for randomized trials (RoB 2) to assess any bias in the randomization process, any deviation from the intended intervention, missing outcome data, measurement of the outcome, selection of reported results, and overall risk of bias. The ROBINS-I Risk of Bias in Non-Randomized Studies - of Interventions was used to assess bias due to confounding, selection of participants into the study, classification of interventions, deviations from intended interventions, missing data, and measurement of measurement outcomes, selection of the reported result. The National Institute of Health (NIH) quality assessment tools were used for the before-after study with no control group for single-arm studies. Seven reviewers, SRP, KG, AD, SS, SB ^1^, Bibh. A, and SB ^2^ were involved in the risk of bias assessment.

## Results

### Studies included

**Figure 1.**
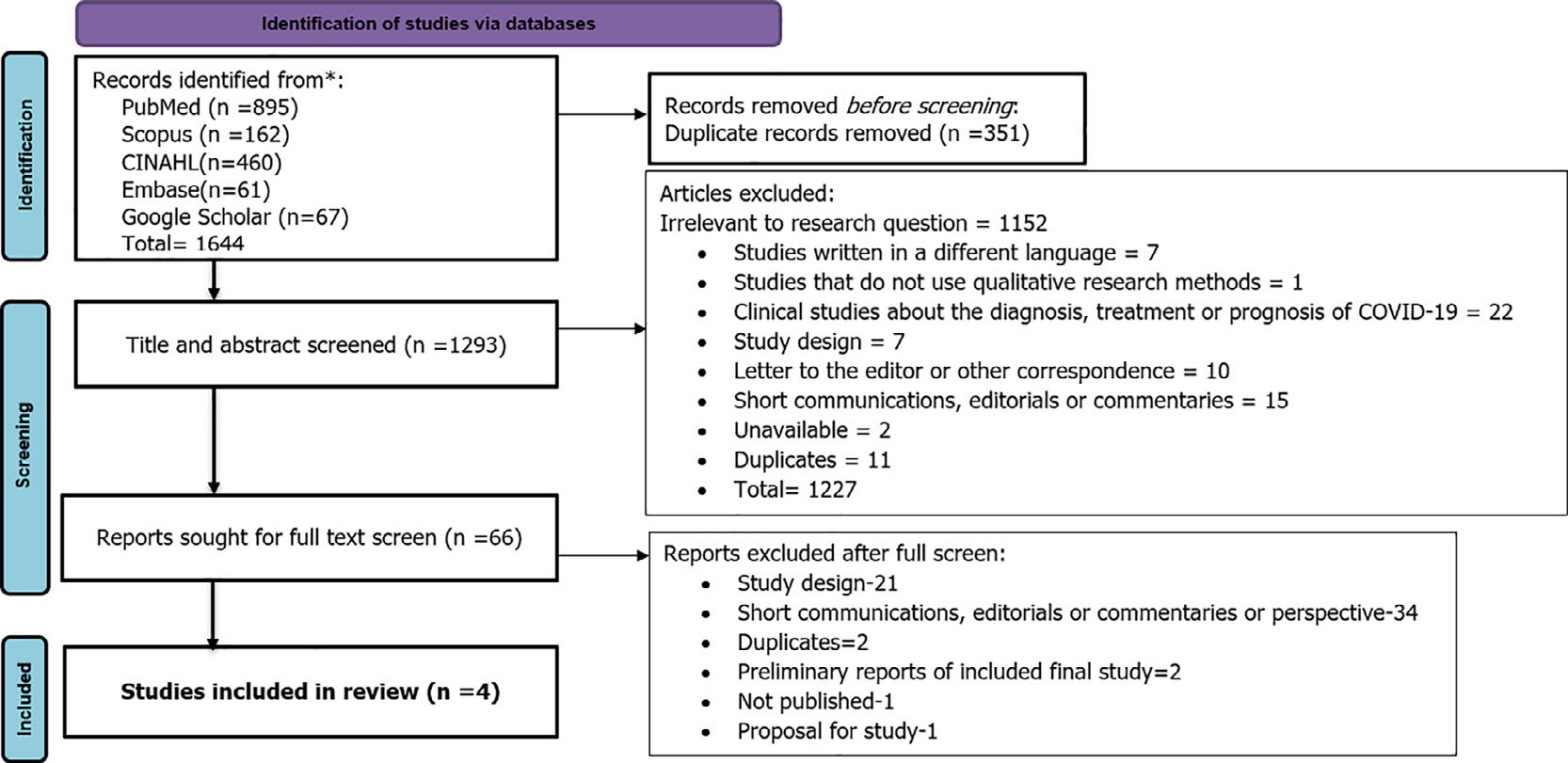
Preferred Reporting Items for Systematic Reviews and Meta-Analyses (PRISMA) flow diagram.

The search using the appropriate terms yielded 1644 potentially relevant articles from Pubmed, Scopus, CINAHL, Google Scholar, and Embase. Through the database search, we found 1644 studies, and 351 among them were identified as duplicates and removed. We screened 1293 studies with titles and abstracts and excluded 1227 studies among them. Then the remaining 66 studies were thoroughly assessed for full-text eligibility. Finally, a total of four studies were listed for the qualitative analysis. This information is visually presented in the PRISMA flow diagram ( Figure 1).

### Study characteristics

Sanmamed *et al*. (2021) studied the effects of 100 cGy radiotherapy on total lungs in a single fraction on patients who were COVID-19 positive, at phase two or three by lung involvement or oxygen requirement. The authors suggested that low-dose radiotherapy could also be a treatment option even after 14 days if the anti-COVID treatment fails. However, there is a possible confounding effect of prior anti-COVID therapy with steroids, hydroxychloroquine, lopinavir/ritonavir, tocilizumab, or remdesivir in this study. The limited number of patients and it being a single-arm study are other limitations. ^31^

A trial conducted in Georgia, USA (Hess *et al*., 2021) studied the effects of 1.5 Gy radiotherapy on both lungs of COVID-19 patients. They selected ten patients that were oxygen-dependent and non-intubated. The control group was chosen from a separate trial conducted in another institution among COVID-19 positive patients. They were matched with the intervention group by age and comorbidities and were given supportive treatment with or without anti-COVID drug therapy. Four patients in the intervention group received azithromycin, one received steroids, and all ten received primary supportive care; hence, the study has possible confounding effects. ^33^

In Tehran, Iran, Ameri *et al*., 2021 studied the effects of 0.5 or 1.0 Gy single fraction low-dose whole-lung irradiation (LD-WLI) in 10 patients with moderate COVID-19 pneumonia. Five patients received 0.5 Gy single fraction radiotherapy, four received 1 Gy single fraction radiotherapy, and one received 0.5 Gy radiation twice; a second therapy was given after clinical deterioration following the first few days of improvement. ^34^

Researchers in Switzerland (Papachristofilou *et al*., 2021) conducted a double-blinded randomized controlled trial in which 22 patients were randomized in two groups with 11 patients in each, receiving 1 Gy low-dose radiation vs. sham radiation. The study did not significantly improve ventilator-free days after 15 days, overall survival, PaO2/FIO2 ratio, and inflammatory markers when compared among two groups. The lymphocyte reduction was significant in the low-dose radiation group in comparison. The authors indicated no role of low-dose radiotherapy in treating COVID-19 pneumonia. ^5^ The major study characteristics are tabulated in Table 1.

**Table 1.** Baseline characteristics of the included studies.

No.	Author/publication year	Study design	Study site	Number of patients (N)	Patient demographics	Intervention
1	Sanmamed *et al*. (2021) ^31^	Prospective, single-arm, phase 1/2 clinical trial	Servicio de Oncología Radioterápica. Hospital Clínico San Carlos Madrid, Spain	9 (single arm study)	The median age was 66 (interquartile range, 57–77); Male 68 yrs required domiciliary O _2_.	100 cGy (Centigray) to total lungs in a single fraction
2	Hess *et al*. (2021) ^33^	Investigator-initiated, single-institution combined phase 1 and 2 trial	Emory University Hospital Midtown/Winship cancer institute; Emory Saint Joseph’s Hospital	20 (10 in intervention and 10 in the control group)	The median age was 78 (43–104) and 75 (44–99) for the LDRT and control cohorts.	1.5 Gy whole-lung low-dose radiotherapy
3	Ameri *et al*. (2021) ^34^	Single-arm pilot trial	Shahid Beheshti University of Medical Sciences, Tehran, Iran	10 (single arm)	The median age was 75 years (range, 60–87 years)	0.5–1 Gy radiotherapy.
4	Papachristofilou *et al*. (2021) ^5^	Randomized double-blind study	University Hospital of Basel in Basel, Switzerland; ICU	22 (11 in intervention and 11 in the control group)	Median of 75 years old (range, 54–84)	1 Gy whole-lung LDRT or sham radiation therapy (sham-RT)

### Risk of bias

**Figure 2.**
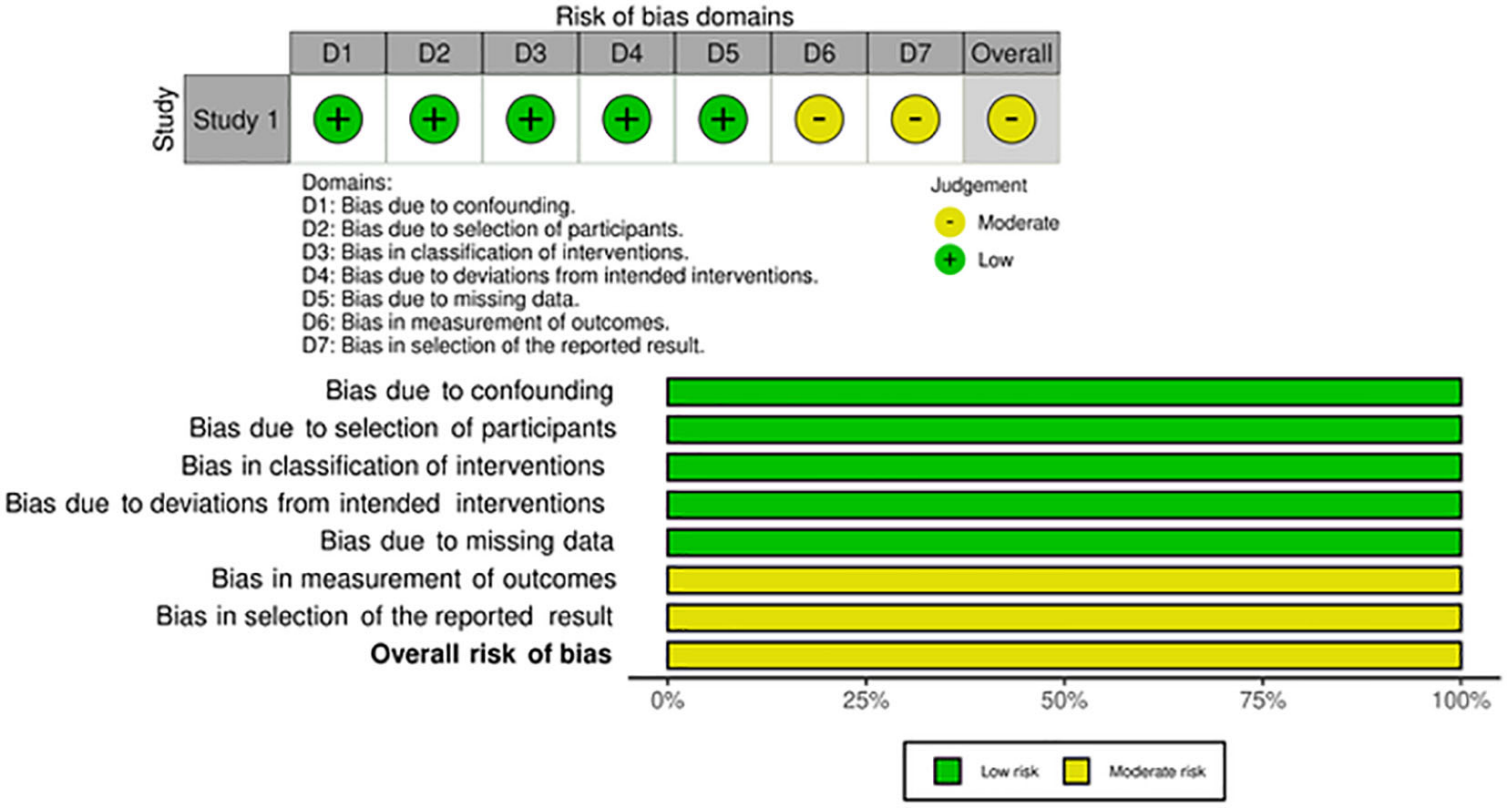
Risk of bias assessment of study two by Hess *et al*. ^33^

**Figure 3.**
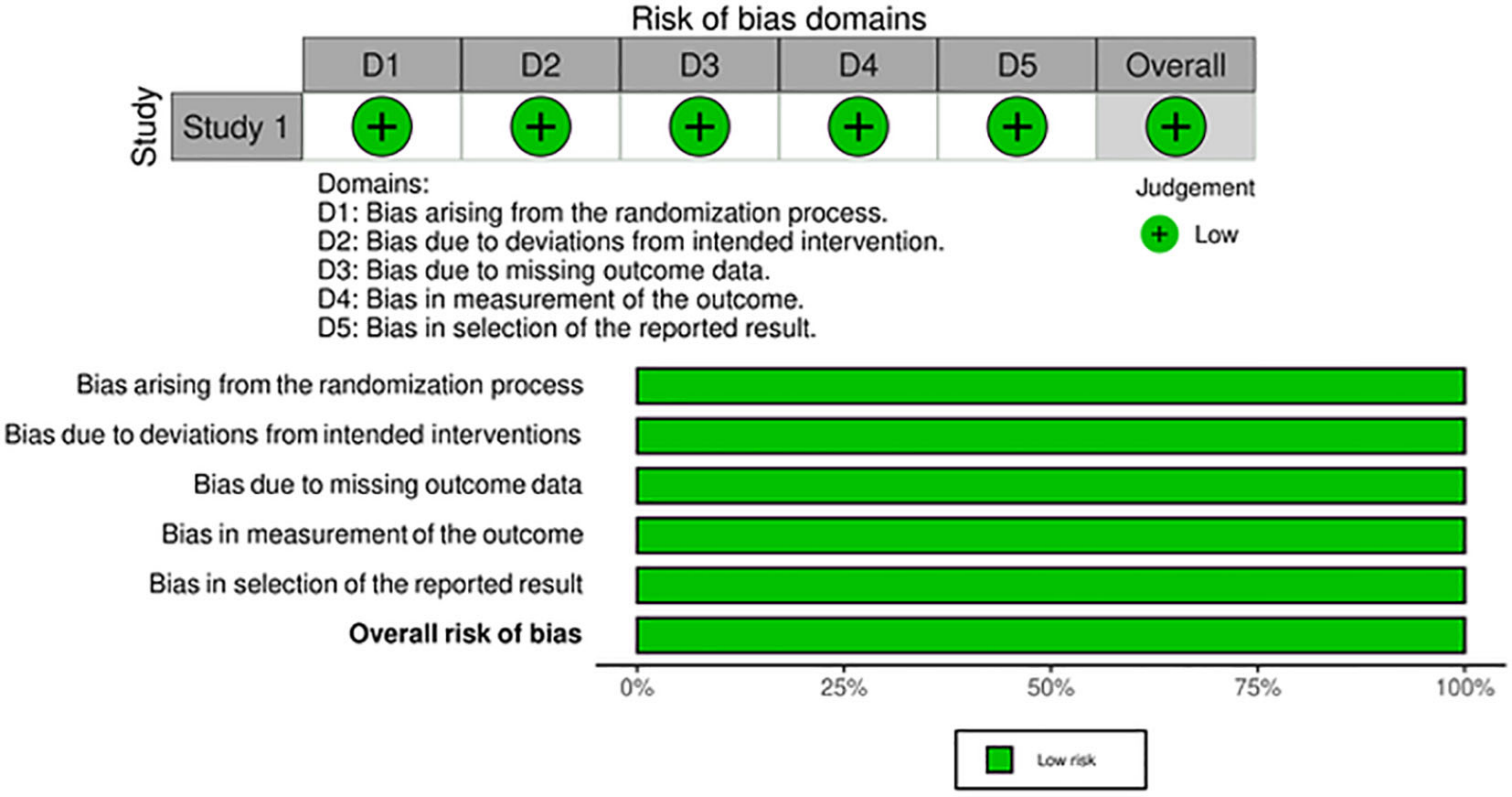
Risk of bias assessment of study four by Papachristofilou *et al*. ^5^

We included four studies, which had different study designs. Therefore, we applied different risk of bias assessment tools for different studies. Studies one (Sanmamed *et al*., 2021) and three (Ameri *et al*., 2021) were single-arm before and after studies, ^31^^,^^34^ study two (Hess *et al*., 2021) was quasi-experimental, ^33^ and study four (Papachristofilou *et al*., 2021) was an RCT. ^5^ For studies one and three, we used the National Institute of Health (NIH) quality assessment tools for before-after studies with no control group for single-arm studies. According to the NIH quality assessment tool, we found both studies to have fair risk of bias. The assessments of Sanmamed *et al*., ^31^ and Ameri *et al*., ^34^ are presented in Table 2. For study 2 (Hess *et al*., 2021), we used the Risk of Bias in Non-Randomized Studies - of Interventions (ROBINS-I) ^37^ and found the study to have a moderate risk of bias. We generated a traffic light plot and summary of the assessment, presented in Figure 2. In study 4 (Papachristofilou *et al*., 2021), we used the Cochrane risk-of-bias tool for randomized trials version 2 (RoB 2). ^5^ We found the study to have a low risk of bias ( Figure 3). ^32^

**Table 2.** Risk of bias assessment of study 1 by Sanmamed *et al*. ^31^ and study 3 by Ameri *et al*. ^34^ carried out according to National Institute of Health quality assessment tools for before-after study with no control group.

Questions for Assessment	YES/NO/Others (CD, NR, NA) *	
Was the study question or objective clearly stated?	YES	YES
Were eligibility/selection criteria for the study population prespecified and clearly described?	YES	YES
Were the participants in the study representative of those who would be eligible for the test/service/intervention in the general or clinical population of interest?	YES	YES
Were all eligible participants that met the prespecified entry criteria enrolled?	NO	YES
Was the sample size sufficiently large to provide confidence in the findings? Was the test/service/intervention clearly described and delivered consistently across the study population?	NO	NO
Was the test/service/intervention clearly described and delivered consistently across the study population?	YES	YES
Was the outcome measures prespecified, clearly defined, valid, reliable, and assessed consistently across all study participants?	YES	NO
Were the people assessing the outcomes blinded to the participants’ exposures/interventions?	NR	NO
Was the loss to follow-up after baseline 20% or less? Were those lost to follow-up accounted for in the analysis?	NO, CD	YES, YES
Did the statistical methods examine changes in outcome measures from before to after the intervention? Were statistical tests done that provided p values for the pre-to-post changes?	YES, YES	YES, YES
Were outcome measures of interest taken multiple times before the intervention and multiple times after the intervention (i.e., did they use an interrupted time-series design)?	NR, YES	NR, YES
If the intervention was conducted at a group level, did the statistical analysis take into account the use of individual-level data to determine effects at the group level?	NO	NO
Overall	FAIR	FAIR

* CD (Cannot determine); N.A. (Not applicable); N.R. (Not reported).

### Results of individual studies

In study one (Sanmamed *et al*., 2021), there was a decrease in the acute phase reactants after one week of radiotherapy but compared to baseline, only lactate dehydrogenase (LDH) showed a significant decline (P = 0.04). The preliminary report also showed significant improvement in the SatO _2_/FiO _2_ index (SAFI). Seven patients were discharged, maintaining supplemental oxygen (maximum 3L). ^31^

The intervention group showed earlier clinical recovery and discharge in study two (Hess *et al.*, 2021). The 28-day overall survival had no significant difference, while freedom from intubation was 90% vs. 60% (P = 0.16). Lower oxygen requirement at the time of intervention and less time to clinical recovery (P = 0.01) were needed by patients aged 65 or over in the intervention group. Still, no significant changes in the control group were observed. Neither group showed significant improvement in radiographic imaging. The study showed a significant reduction in inflammatory biomarkers (C-reactive protein (CRP) and LDH), cardiac markers, white blood cells. There was a considerable lack of leukocytosis in the intervention group compared to the control group. Although not significant, the study suggested the possibility of prevention of elevation of hepatic biomarkers by low-dose radiotherapy. ^33^

In study three (Ameri *et al*., 2021), the overall clinical recovery shown by the study was 60%. Still, there was no significant improvement in clinical recovery and SpO _2_ among both groups of radiation dose patients. In the trial, four patients died before discharge, and two died immediately after being discharged. Patients who survived showed some improvement in the inflammatory markers. ^34^

In study four (Papachristofilou *et al*., 2021), the study did not significantly improve ventilator-free days after 15 days, overall survival, PaO _2_/FIO _2_ ratio, and inflammatory markers when compared among the two groups. The lymphocyte reduction was significant in the low-dose radiation group in comparison. ^5^

The major outcomes of the review are summarized in Table 3, where descriptive comparative data are presented as the same measures were not followed throughout all four studies.

**Table 3.** Individual study results.

No.	Outcomes					Toxicity (N)	Reduction in inflammatory markers (ESR, CRP, IL6, D-dimer, ferritin, etc.)	Concomitant interventions
	Primary		Secondary					
	Deaths (N)	Discharge(N)	Oxygen status	Mechanical ventilation required	CT-scan findings			
Sanmamed *et al*. (2021) ^31^	Two	Seven	Seven patients presented with baseline Severe Respiratory Failure (SRF) and 2 with Mild Respiratory Failure (MRF) Seventy-two hours after R.T., there was a significant improvement (P = 0.01): 2 patients continued with SRF, 3 patients with MRF and four patients recovered normal SAFI (Oxygen saturation/Inspired fraction of oxygen) Index. A week later, the significant improvement persisted (P = 0.01), One patient continued with SRF, 3 with MRF, and 5 recovered normal SAFI	Not required (at discharge)	Significant improvement on day 7 (P = 0.03)	GRADE 2 Lymphopenia (2) One patient with baseline Grade 3 worsened to Grade 4 one week after R.T.	Reduced. Only LDH reduced significantly. (P = 0.04)	Prednisone or methylprednisolone, Hydroxychloroquine, Lopinavir/ritonavir Tocilizumab Remdesivir Antithrombotic
Hess *et al*. (2021) ^33^	One death in each group (One in intervention, one in control)	Nine in each group	Median P: F ratio: ratio of arterial pressure (mm Hg) of oxygen (PaO2) to a fraction of inspired oxygen (FiO2) (range) Intervention-138 (79–281), Control-194 (100–452), Combined- 171 (79–452) (P = 0.25)	One patient in intervention and four patients in control group required intubation after intervention.	Any radiographic improvement by day 21 occurred in 90% Vs 57% of patients in the LDRT compared to control cohorts (P = 0.12) Intervention group- improvement in 9; Control group-improvement in 4.	Reduced monocytes and Neutrophil to Lymphocyte ratio. Upper G.I. acute toxicity in 1 patient.	Significant reduction.	IV hydrocortisone azithromycin, Hydroxychloroquine, Prednisone, remdesivir.
Ameri *et al*. (2021) ^34^	Four	6 (2 patients among discharged died after being discharged- after one day and three days respectively.)	The mean magnitude of the improvement in SpO2 at days 1 and 2 after R.T. was 2.4% (4.8%) and 3.6% (6.1%), respectively. In the 0.5 and 1.0 Gy groups, the mean improvement in SpO _2_ within two days was 6.1 versus 0.25% (P = 0.95), respectively.	Not required.	NA	NA	Not significant.	Not received.
Papachristofilou *et al*. (2021) ^5^	Six in the intervention group; five in the control group (Sham irradiation)	Four patients in each group were discharged.	no significant differences were seen in oxygenation changes within 24 hours (LDRT vs. sham-RT: median PaO _2_/FIO _2_ change + 5 vs. + 9, P = 0.49)	Mechanical ventilation free days for 4-4 patients in each group after 15 days.	NA	Reduced lymphocyte count.	No significant difference between groups.	Dexamethasone Remdesivir Experimental drug

ESR: erythrocyte sedimentation rate; CRP: C-reactive peptide; D-dimer: domain-dimer; LDH: lactate dehydrogenase.

## Discussion

Two of our included studies indicated improvement in inflammatory markers to some extent with low dose radiotherapy, but these studies also prescribed steroids or other drugs like azithromycin, hydroxychloroquine, tocilizumab, remdesivir to patients ^31^^,^^33^; the two other studies did not show any significant improvement. ^5^^,^^33^ LDRT has been used to treat various acute and chronic inflammatory diseases since 1900; Musser and Edsall introduced radiotherapy to treat pneumonia in 1905. ^35^ The mechanism of single LDRT (0.2 to 1.0) proved to be highly effective in treating pneumonia. It involved the induction of an anti-inflammatory phenotype that led to rapid clinical improvements and markedly reduced mortality risk. ^15^ COVID-19 patients with severe pulmonary diseases have increased expressions of inflammatory markers such as C-reactive protein, ferritin, elevated D dimers, and proinflammatory cytokines. ^36^ In such cases, low-dose radiation therapy could provide a therapeutic arsenal against COVID-19-related complications and associated morbidity and mortality. ^37^ COVID with ARDS requires oxygen and ventilatory support, yet mortality during mechanical ventilation is high despite such measures. ^36^ COVID-19 can cause inflammation in the lungs, hypoxemia, and increased breathing. Patients may need early intubation, further damaging the lungs. ^38^ In such cases, several inflammatory conditions, including bacterial/viral pneumonia, have been successfully treated with radiation therapy. ^39^

The study by Sanmamed *et al*. showed significant improvement in extension score between the first simulation computed tomography (CT) scan and day seven CT-scan after radiotherapy (p = 0.03). Still, no significant difference was found in severity score by imaging. ^31^ Other studies did not follow nor study CT-scan changes homogeneously before and after radiotherapy. ^5^^,^^33^^,^^34^ Hence, our review could not determine if LDRT plays any role in bringing significant changes/improvement in C.T. chest findings in COVID-19 pneumonia. The features of the chest C.T. of COVID patients depend on scanning time, age of the patient, the condition of disease during follow-up, immune status of the patient, drug therapy provided, and the underlying pathology. ^40^ The most common findings of C.T. are ground-glass opacity at peripheral and lower lobes, patchy consolidations in multiple areas with the peripheral and central distribution. ^40^^,^^41^

Three of the included studies showed reduced white blood cells, especially lymphocytes, essential for resistance against COVID-19. These three studies also used other anti-COVID drugs like steroids such as dexamethasone, remdesivir, or monoclonal antibodies. ^5^^,^^31^^,^^33^ Dexamethasone can cause lymphopenia, ^42^ but our review has no concrete evidence as to what caused the reduction of white blood cells. Remdesevir has shown an improved rate of clinical recovery in COVID-19 patients. ^43^

Studies regarding the toxicity of LDRT suggest that doses lower than 1 Gy may not majorly concern short-term or long-term follow-up. ^39^ The risk of radiation injury in medical imaging has been discussed in the past and hovers between 10 mSv to 100 mSv. ^44^ but, according to the 2006 BEIR VII lifetime attributable cancer risk model, 1 in 1000 can develop cancer with a radiologic procedure dose of 10 mSv. ^45^ However, evidence suggests that the radiation dose of 0.15–1.5 Gy is linearly related to solid cancer induction (i.e., a range of approximately 1 log). ^46^ Ameri *et al*. suggest LDRT (<1 Gy) can yield anti-inflammatory effects, while more than 1 Gy can enhance the proinflammatory development and requires more extensive study. ^34^ Various experiments in cats and mice exposed to 0.5–1 Gy 24h after virus inoculation showed a beneficial protective effect. ^39^ Treatment with LDRT suggests improved cytokine release syndrome, a significant reduction in total leukocyte counts, serum creatinine, serum liver enzymes, alanine aminotransferase (ALT), and aspartate aminotransferase (AST). ^39^ For COVID-19 patients that present with a cytokine storm, a single total dose of 0.3–0.5 Gy targeted radiotherapy is beneficial in reducing the possibility of any immediate or long-term adverse effects. ^24^

A recovery trial showed lower 28-day mortality among those receiving invasive mechanical ventilation or oxygen alone at randomization with dexamethasone. ^13^ Hence, this steroid is a proven medication with a mortality benefit at the moment. However, Dexamethasone also has several side effects like hormonal imbalance, weight gain, fluid retention, anxiety, disturbed sleep patterns, withdrawal symptoms, etc. There is a risk of fungal infection, as recently seen with a rise in Mucormycosis cases in India ( steroid side effects) ^47^ Thus, having LDRT as a treatment option for those who are not ideal candidates for steroids could prove to be a boon.

### Limitations

The studies included in this review have a limited number of patients, mainly assessed in a single facility. They cannot, therefore, truly determine the actual effect on the general population. The included studies also used different methods, including two single-arm studies, one RCT, and one non-randomized (Quasi-experimental) study. ^5^^,^^31^^,^^33^^,^^34^ Although two studies showed some improvement regarding inflammatory markers, and one showed improvement in extension score before concluding, we must also consider the possibility of confounding effects by using drugs capable of anti-inflammatory effect dexamethasone and remdesivir. ^43^^,^^48^^,^^49^ All the included studies do not have thorough follow-up information about ventilator use and their outcome either, so it is hard to make any firm conclusions about how LDRT can bring about changes to lung status during COVID pneumonia.

## Conclusion

Only two studies included in this review demonstrated an improvement in inflammatory markers; however, patients were also given steroids or other drugs. Therefore, the confounding effects must be considered. This systematic review does not support any clinical benefit from LDRT. As of now, this systematic review of the available literature does not provide sufficient evidence to back up any mortality benefit, improvement in the clinical course, or imaging changes with LDRT. As a possible alternative treatment, we suggest large-scale studies with proper dose calculations and greater vigilance of the short-term and long-term beneficial effects and toxicity.

## Data availability

### Underlying data

Figshare: Underlying data for ‘Effectiveness of low-dose radiation therapy in COVID-19 patients globally: A systematic review’ https://doi.org/10.6084/m9.figshare.c.5757326.v1 ^32^

This project contains the following underlying data:

• Data Extraction
• Search strategy and authors role
• PubMed search details
• Risk of bias assessment of study 1 and 3 (word file created following the NIH quality assessment tool guideline)
• Risk of bias assessment of study 2 (ROBIN-I)
• Risk of bias assessment for study 4 (ROB-2)

## Reporting guidelines

Figshare: PRISMA checklist for ‘Effectiveness of low dose radiation therapy in COVID-19 patients globally: A systematic review’. https://doi.org/10.6084/m9.figshare.c.5757326.v1 ^32^

Data are available under the terms of the Creative Commons Attribution 4.0 International license (CC-BY 4.0)
