# Supplementary material for: Effectiveness of low-dose radiation therapy in COVID-19 patients globally: A systematic review
Source: F1000Res. 2022 Jan 19;11:62. [Version 1] doi: 10.12688/f1000research.74558.1 (PMC8825648; doi:10.12688/f1000research.74558.1)
Supplement: Peer review history

# Peer review: Reviewer response for version 1

**DOI:** 10.5256/f1000research.78329.r120360

**Published:** 2022-02-08

**Authors:** Prashant Gupta

**Affiliations:** 1 Department of Radiology, National Academy of Medical Sciences, Bir Hospital, Kathmandu, Nepal

The authors have done a great job to evaluate the role of low-dose radiotherapy in the management of COVID-19 patients. However, the included studies' number is small which has underpowered this review and may have an effect on the conclusion. Methods, analysis, and presentation of data need to be described in a little bit more detail and in a more clarified way. They have well-described about the biases and confounders about the included studies in this review that have caused limitations in the study.

 Specific recommendations are as follows:

 1. In Table 2, the answers column are not clearly defined for which group.

 2. Much information in Table 3 is not written clearly. I missed the flow in writing.

 3. Data extraction portion could be written more clearly.

 4. In the methods section, eligibility criteria need to be revised too.

Are the rationale for, and objectives of, the Systematic Review clearly stated?

Yes

Is the statistical analysis and its interpretation appropriate?

Partly

Are sufficient details of the methods and analysis provided to allow replication by others?

Partly

Are the conclusions drawn adequately supported by the results presented in the review?

Partly

Reviewer Expertise:

Diagnostic Radiology, Radio-interventions, Clinical research

I confirm that I have read this submission and believe that I have an appropriate level of expertise to confirm that it is of an acceptable scientific standard, however I have significant reservations, as outlined above.

# Peer review: Reviewer response for version 1

**DOI:** 10.5256/f1000research.78329.r120357

**Published:** 2022-01-25

**Authors:** Ritesh Neupane

**Affiliations:** 1 Department of Medicine, Penn State Health Milton S Hershey Medical Center, Hershey, PA, USA

The review attempts to look at an interesting modality of treatment possibility for COVID-19 patients: LDRT. The methods are clear and appear rigorously done. It concludes that, despite improvement in inflammatory markers, LDRT cannot be sufficiently supported due to strong confounding factors like use of steroids and other currently approved therapies. The conclusion goes in tandem with the observations, though it is quite underpowered.

 I would recommend authors and co-authors highlight why this review work is different from the paper by Kapoor et al which has evaluated the studies presented in this submitted report.

Are the rationale for, and objectives of, the Systematic Review clearly stated?

Yes

Is the statistical analysis and its interpretation appropriate?

Partly

Are sufficient details of the methods and analysis provided to allow replication by others?

Yes

Are the conclusions drawn adequately supported by the results presented in the review?

Partly

Reviewer Expertise:

Clinical research in infectious disease

I confirm that I have read this submission and believe that I have an appropriate level of expertise to confirm that it is of an acceptable scientific standard, however I have significant reservations, as outlined above.

## References

[1] : Low-dose radiation therapy (LDRT) for COVID-19 and its deadlier variants. *Archives of Toxicology* .2021;95(10) : 10.1007/s00204-021-03124-x 3425-3432 10.1007/s00204-021-03124-x PMC830808134302492

# Peer review: Reviewer response for version 1

**DOI:** 10.5256/f1000research.78329.r120350

**Published:** 2022-01-24

**Authors:** Sudeep Acharya

**Affiliations:** 1 Department of Internal Medicine, Staten Island University Hospital, New York, NY, USA

The authors have a good research question which falls within current literature. They have clearly described the methods of data collection and interpretation. This is a small study and very small population group analysed which might have resulted in underpowered study. Also there were many variables and confounders in those analysed studies which the authors have mentioned in the limitation part. Overall they have done a good job trying to look for an alternative pathway in the treatment of COVID-19 when there is no certain guideline to treat one.

Are the rationale for, and objectives of, the Systematic Review clearly stated?

Yes

Is the statistical analysis and its interpretation appropriate?

I cannot comment. A qualified statistician is required.

Are sufficient details of the methods and analysis provided to allow replication by others?

Yes

Are the conclusions drawn adequately supported by the results presented in the review?

Yes

Reviewer Expertise:

Infectious disease, pulmonary disease, critical care medicine, mechanical ventilation.

I confirm that I have read this submission and believe that I have an appropriate level of expertise to confirm that it is of an acceptable scientific standard.
